# Supplementary material for: Heat adaptation of phage T7 under an extended genetic code
Source: Virus Evol. 2021 Dec 1;7(2):veab100. doi: 10.1093/ve/veab100 (PMC8923235; doi:10.1093/ve/veab100)
Supplement: veab100_Supp [file veab100_supp.zip › Sequencing_Analysis.zip!/Sequencing_Analysis/A1/evidence/JC_SIDE_2_52.html]

BRESEQ :: Evidence


| Predicted mutation | | | | | | |
| --- | --- | --- | --- | --- | --- | --- |
| evidence | seq id | position | mutation | annotation | gene | description |
| MC JC | NC\_001604.1 | 1,257 | Δ1,480 bp | intergenic (–/–) | – / – | –/– |

| Missing coverage evidence... | | | | | | | | | | |
| --- | --- | --- | --- | --- | --- | --- | --- | --- | --- | --- |
|  |  |  | seq id | start | end | size | ←reads | reads→ | gene | description |
| \* | \* | ÷ | NC\_001604.1 | 1257 | 2736 | 1480 | 3465 [0] | [3] 3461 | *–/–* | –/– |

| New junction evidence | | | | | | | | | | | |
| --- | --- | --- | --- | --- | --- | --- | --- | --- | --- | --- | --- |
|  | | seq id | position | reads (cov) | reads (cov) | score | skew | freq | annotation | gene | product |
| \* | ? | NC\_001604.1 | = 1256 | 0 (0.000) | 3355 (1.140) | 393/574 | 0.0 | 100% | intergenic (–/–) | *–/–* | –/– |
|  | ? | NC\_001604.1 | 2737 = | 0 (0.000) |  |  |  |  | intergenic (–/–) | *–/–* | –/– |

Only 100 of 3461 total aligned reads displayed.  

`ACAATGATGCCCATTATAAATACGCTGAGATTGCAAGCGACATCATTGATTGCAATTCGGATGAGCATGATGAGTTAACTGGATGGGATGGTGAGTTTGTTGAAACTTGTAAACTAATCCGCAAGTTCTTTGAGGGCATCGCCTCATTCGACATGCATAGCGGGAACATCATGTTCTCAAATGGAGACGTACCATACATCACCGACCCGGTATCATTCTCGCAGAAGAAAGACGGTGGCGCATTCAGCATCGACCCTGAGGAACTCATCAAGGAAGTCGAGGAAGTCGCACGACAGAAAGAAATTGACCGCGCTAAGGCCCGTAAAGAACGTCACGAGGGGCGCTTAGAGGCACGCAGATTCAAACGTCGCAACCGCAAGGCACGTAAAGCACACAAAGCTAAGCGCGAAAGAATGCTTGCTGCGTGGCGATGGGCTGAACGTCAAGAACGGCGTAACCATGAGGTAGCTGTAGATGTACTAGGAAGAACCAATAACGCTATGCTCTGGGTCAACATGTTCTCTGGGGACTTTAAGGCGCTTGAGGAACGAATCGCGCTGCACTGGCGTAATGCTGACCGGATG  >  NC_001604.1/2454‑3037`  

                                                                                                                                                                                                                                                                                           `|`                                                                                                                                                                                                                                                                                                              
`aaatgctgaaagaaaacatcctttatgatgacatccgtgacactgatgccctgcacgatgctattcacatggctgccggtaatgcagttccgcactactacgctgacatctttagcgtaatggcaagtgagggcattgaccttgagttcgaagactctggtctgatgcctgacaccaaggacgtaatccgcatcctgcaagcgcgtatctatgagcaattaacgattgacctctgggaagacgcagaagacttgctcaatgaatacttggaggaagtcgaggaAGTCGCACGACagaaaga                                                                                                                                                                                                                                                                                             <  1:6595‑M2/18‑1 (MQ=255)`  
`aaatgctgaaagaaaacatccgttatgatgacatccgtgacactgatgacctgcacgatgctattcacatggctgccgataatgcagttccgcactactacgctgacatctttagcgtaatggcaagtgagggcattgaccttgagttcgaagactctggtctgatgcctgacaccaaggacgtaatccgcatcctgcaagcgcgtatctatgagcaattaacgattgacctctgggaagacgcagaagacttgctaaatgaatacttggaggaagtcgaggaAGTCGCACGACagaaaga                                                                                                                                                                                                                                                                                             <  1:344277‑M2/18‑1 (MQ=255)`  
         `aagaaaacatacgttatgatgacatccgtgacactgatgacctgctcgatgctattcacatggctgccgataatgcagttccgaactactacgctgacatctttagcgtaatggcaagtgagggcattgaccttgagttcgaagactctggtctgatgcctgacaccaaggacgtaatccgcatcctgcaagcgcgtatctatgagcaattaacgattgacctctgggaagacgcagaagacttgctcaatgaatacttggaggaagtcgaggaAGTCGCACGACAGAAAGAAATTGACcg                                                                                                                                                                                                                                                                                    <  1:173256‑M2/27‑1 (MQ=255)`  
                  `tgtgtataagagacagccgtgacactgatgacctgcacgatgctattcacatggctgccgataatgcagttccgaactactacgctgacatctttagcgtaatggcaagtgagggcattgaccgtgagttcgaagactctggtctgatgcctgacaccaaggacgtaatccgcatcctgcaagcgcgtatctatgagcaattaacgattgacctctgggaagacgcagaagacttgctcaatgaatacttggaggaagtagaggaAGTCGCACGACAGAAAGAAATTGACCGCGCTAAGGc                                                                                                                                                                                                                                                                           <  1:362266‑M2/36‑1 (MQ=255)`  
                  `tccgttatgatgacatccgggatactgatgacctgaccgatgctattcacatggctgccgataatgcagttccgcactactacgctgacatctttagcgtaatggcaagtgagggcattgaccttgagttcgaagactctggtctgatggctgacaccaaggacgtaatccgcatcctgcaagcgcgtatctatgagcaattaacgattgacctctgggaagacgcagaagacttgctcaatgaatacttggaggaagtcgaggaAGTCGCACGACAGAAAGAAATTGACCGCGCTAAGGc                                                                                                                                                                                                                                                                           <  1:476800‑M2/36‑1 (MQ=255)`  
                   `gagacatgatgtcatccgtgacactgatgacccgcacgatgctattcacatggctgccgataatgcagttccgcacgactacgctgacatctttagcgtaatggcaagtgagggcattgaccttgagttcgaagactctggtctgatgcctgacaccaaggacgtaatccgcatcctgcaagcgagtatctatgagcaattaacgattgacctctgggaagacgcagaagacttgctcaatgaatacttggaggaagtcgaggaAGTCGCACGACAGAAAGAAATTGACCGCGCTAAGGcc                                                                                                                                                                                                                                                                          <  1:247858‑M2/37‑1 (MQ=255)`  
                        `attatgacatccgttacactgatgaccggcacgatgctattcaaagggctgccgataatgcatgtccgcactactacgctgacatctttagcgtaatggcaagtgagggcattgaccttgagttcgaagactctggtctgatgcctgacaccaaggacgtaatccgcatcctgcaagcgcgtatctatgagcaattaacgattgacctctgggaagacgcagaagacttgctcaatgaatacttggaggaagtcgaggaAGTCGCACGACAGAAAGAAATTGACCGCGCTAAGGCCCGTaa                                                                                                                                                                                                                                                                     <  1:386048‑M2/42‑1 (MQ=255)`  
                            `ggacagccgtgaccctgatgacctgcacgatgctattcacatggctgccgttaatgcagttccgcactactacgctgacatctttagcgtaatggcaagtgagtgcattgaccttgagttcgaagactctggtctgatgcctgacacaaaggacgtaatccgcatcctgcaatcgcgtatctatgagcaattaacgattgacctctgggaagacgcagaagacttgctcaatgaatacttgggggaagtcgaggaAGTCGCACGACAGAAAGAAATTGACCGCGCTAAGGCCCGTAAAGaa                                                                                                                                                                                                                                                                 <  1:290387‑M2/46‑1 (MQ=255)`  
                                  `gatgtgtataagagacaggcacgatgctattcacatggctgccgataatgcagttccgcactactacgctgacatctttagcgtaatggcaagtgagggcattgaccttgagttcgaagactctggtctgatgcctgacaccaaggacgtaatccgcatcctgcaagcgcgtatctatgagcaattaacgattgacctctgggaagacgcagaagacttgctcaatgaatacttggaggaagtcgaggaAGTCGCACGACAGAAAGAAATTGACCGCGCTAAGGCCCGTAAAGAACGTCAc                                                                                                                                                                                                                                                           <  1:429025‑M2/52‑1 (MQ=255)`  
                                  `ccgtgacactgatgacctgcacgatgctattcacatggctgccgataatgcagttccgcactactacgctgacatctttagcgtaatggcaagtgagggcattgaccttgagttcgaagactctggtctgatgcctgacaccaaggacgtaatccgcatcctgcaagcgcgtatctatgagcaattaacgattgacctctgggaagacgcagaagacttgctcaatgaatacttggaggaagtcgaggaAGTCGCACGACAGAAAGAAATTGACCGCGCTAAGGCCAGTAAAGAACGTAAc                                                                                                                                                                                                                                                           >  1:166752‑M2/250‑301 (MQ=255)`  
                                      `ctcggagatgtgtataagagacagattcacatggctgccgataatgcagttccgcactactacgctgacatctttagcgtaatggcaagtgagggcattgaccttgagttcgaagactctggtctgatgcctgacaccaaggacgtaatccgcatcctgcaagcgcgtatctatgagcaattaacgattgacctctgggaagacgcagaagacttgctcaatgaatacttggaggaagtcgaggaAGTCGCACGACAGAAAGAAATTGACCGCGCTAAGGCCCGTAAAGAACGTCACGAgg                                                                                                                                                                                                                                                       <  1:189294‑M2/56‑1 (MQ=255)`  
                                        `cacgaatgaccggaaggatgctattcaaatggctgcagataatgcagttccgaactactacgatgacatctttagcgtaatggcaagtgagggcattgaccttgagttagaagactctagtctgatgcctgacaccaaggacgtaatccgcatcatgcaagcgcgtatctatgagcaagtaacgattgacctctgggaagacgcagaagacttgctcaatgaatacttggaggaagtcgaggaAGTCGAACGACAGAAAGAAATTGACCGCGCTAAGGCCCGTAAAGAACGTCACGAgggg                                                                                                                                                                                                                                                     <  1:137262‑M2/58‑1 (MQ=255)`  
                                                    `gcacgatgctattcacatggctgccgataatgcagttccgcactactacgctgacatctttagcgtaatggcaagtgagggcattgaccttgagttcgaagactctggtctgatgcctgacaccaaggacgtaatccgcatcctgcaagcgcgtatctatgagcaattaacgattgacctctgggaagacgcagaagacttgctcaatgaatacttggaggaagtcgaggaAGTCGCACGACAGAAAGAAATTGACCGCGCTAAGGCCCGTAAAGAACGTAACGAGGGGCGCTTAGAGgcg                                                                                                                                                                                                                                         >  1:278723‑M2/232‑300 (MQ=255)`  
                                                         `atgctattcacatggctgccgataattcagttccgcactactacgctgacatctttagcgtaatggcaagtgagggcattgaccttgagttcgaagactctggtctgatgcctgacaccaaggacgtaatccgcatcctgcaagcgcgtatctatgagcaattaacgattgacctctgggaagacgcagaagacttgctcaatgaatacttggaggaagtcgaggaAGTCGCACGACAGAAAGAAATTGACCGCGCTAAGGCCCGTAAAGAACGTCACGAGGGGCGCTTAGAGGCACGCAg                                                                                                                                                                                                                                    >  1:377726‑M2/227‑301 (MQ=255)`  
                                                         `atgctattcacatggctgccgataatgcagttccgcactactacgctgacatctttagcgtaatggcaagtgagggcattgaccttgagttcgaagactctggtctgatgcctgacaccaaggacgtaatccgcatcctgcaagcgcgtatctatgagcaattaacgattgacctctgggaagacgcagaagacttgctcaatgaatacttggaggaagtcgaggaAGTCGCACGACAGAAAGAAATTGACCGCGCTAAGGCCCGTAAAGAACGTCACGAGGGGCGCTTAGAGGCACGCAg                                                                                                                                                                                                                                    >  1:119507‑M2/227‑301 (MQ=255)`  
                                                         `atgctatccacatggctgccgataatgcagttccgcactactacgctgacatctttagcgtaatggcaagtgagggcattgaccttgagttcgaagactctggcctgatgcgtgacaccaaggacgtaatccgcatcctgcaagcgcgtatctatgagcaattaacgattgacctctgggaagacgcagaagacttgctcaatgaatacttggaggaagtcgaggaAGTCGCACGACAGAAAGAAATTGACCGCGCTAAGGCCCGTAAAGAACGTCACGAGGGGCGCTTAGAGGCACGCAg                                                                                                                                                                                                                                    <  1:367952‑M2/75‑1 (MQ=255)`  
                                                           `gctattcacatggctgccgataatgcagttccgcactactacgctgacatctttagcgtaatggcaagtgagggcattgaccttgagttcgaagactctggtctgatgcctgacaccagggacgtaatccgcatcctgcaagcgcgtatctatgagcaattaacgattgacctctgggaagacgcagaagacttgctcaatgaatacttggaggaagtcgaggaAGTCGCACGACAGAAAGAAATTGACCGCGCTAaggcccgtaaagaacgtcacgagctgtcccttatccccatctccc                                                                                                                                                                                                                                  >  1:305319‑M2/225‑279 (MQ=255)`  
                                                              `attcacatgtctgccgataatgcagttccgcactactacgctgacatctttagcgtaatggcaagtgagggcattgaccttgagttcgaagactctggtctgatgcctgacaccaaggacgtaatccgcatcctgcaagcgcgtatctatgagcaattaacgattgacctctgggaagacgcagaagacttgctcaatgaatacttggaggaagtcgaggaAGTCGCACGACAGAAAGAAATTGACCGCGCTAAGGCCCGTAAAGAACGTTCCGAGGGGCGCTTAGAGGCACGCAGATCCa                                                                                                                                                                                                                               >  1:5318‑M2/222‑301 (MQ=255)`  
                                                                `tcacatggctgccgataatgcagttccgcactactacgctgacatctttagcgtaattgcaagtgagggcattgaccttgagttcgaagactctggtctgatgcctgacaccaaggacgtaatccgcatcctgcaagcgcgtatctatgagcaattaacgattgacctctgggaagacgcagaagacttgctcaatgaatacttggaggaagtcgaggaAGTCGCACGACAGACAGAAATTGACCGCGCTAAGGCCCGTAAAGAACGTCACGAGgggcgcttagaggctgtctcttataca                                                                                                                                                                                                                             >  1:462488‑M2/220‑288 (MQ=255)`  
                                                                  `acatggctgccgataatgcagttccgcactactacgctgacatctttagcgtaatggcaagtgagggcattgaccttgagttcgaagactctggtctgatgcctgacaccaaggacgtaatccgcatcctgcaagcgcgtatctatgagcaattaacgattgacctctgggaagacgcagaagacttgctcaatgaatacttggaggaagtcgaggaAGTCGCACGACAGAAAGAAATTGACCGCGCTAAGGCCCGTAAAGAACGTCACGAGGGGCGCTTAGAGGCACGCAGATTCAAACg                                                                                                                                                                                                                           >  1:81933‑M2/218‑301 (MQ=255)`  
                                                                         `tgccgataatgcagttccgcactactacgctgacatctttagcgtaatggcaagtgagggcattgaccttgagttcgaagactctggtctgatgcctgacaccaaggacgtaatccgcatcctgcaagcgcgtatctatgagcaattaacgattgacctctgggaagacgcagaagacttgctcaatgaatacttggaggaagtcgaggaAGTCGCACGACAGAAAGAAATTGACCGCGCTAAGGCCCGTAAAGAACGTCACGAGGGGCGCTTAGAGGCACGCAGATTCAAACGTCGCAAc                                                                                                                                                                                                                    <  1:115110‑M2/91‑1 (MQ=255)`  
                                                                          `gccgataatgcagttccgcactactacgctgacatctttagcgtaatggcaagtgagggcattgacctttagttcgaagactctggtctgatgcctgacaccaaggacgtaatccgcatcctgcaagcgcgtatctatgagcaattaacgattgacctctgggaagacgcagaagacttgctcaatgaatacttggaggaagtcgaggaAGTCGCACGACAGAAAGAAATTGACCGCGCTAAGGCCCGTAAATAACGTCACGAGGGGCGCTTAGAGGCACGCAGATTCAAACGTCGCAAcc                                                                                                                                                                                                                   >  1:243057‑M2/210‑301 (MQ=255)`  
                                                                            `cgataatgcagttccgcactactacgctgacatctttagcgtaatggcaagtgagggcattgaccttgagttcgaagactctggtctgatgcctgacaccaaggacgtaatccgcatcctgcaagcgcgtatctatgagcaattaacgattgacctctgggaagacgtagaagacttgcgcaatgaatacttggaggaagtcgaggaAGTCGCACGATAGAAAGAAATTAACCGCGCTAAGGACCGTAAAGAACGTCACGA‑GGGAGCTTAGAGGCACGCCGATTCAAACGTCGCAAccgaa                                                                                                                                                                                                                >  1:503826‑M2/208‑299 (MQ=255)`  
                                                                             `gataatgcagttccgcactactacgctgacatctttagcgtaatggcaagtgagggcattgtccttgagttcgaagcctctggtctgatgcctgacaccaaggacgtaatccgcatcctgcaagcgcgtatctatgagcaattaacgattgacctctgggaagacgcagaagacttgctcaatgaatacttggaggaagtcgaggaAGTCGCACGACAGAAAGAAATTGACCGCGCTAAGGCCCGTAAAGAACGTCACGAGGGGCGATTAGAGGCACGCAGATTCAAACGTCGCAACCGCa                                                                                                                                                                                                                >  1:142652‑M2/207‑301 (MQ=255)`  
                                                                                         `gagatgtgtagaagagacagctttagcgtaagggcaagtgagggcattgaccttgagttcgaagactcggggctgatgcctgacaccaaggacgtaatccgcatcctgcaagcgagtatcaatgagcaagtaacgattgacctctgggaagacgcagaagacttgctcaaggaatacttggaggaagtcgaggaAGTCGCACGACAGAAAGAAATTGACCGCGCTAAGGCCCGTAAAGAACGTCACGAGGGGCGCTTAGAGGCACTCAGATTCAAACGTCGCAACCGCAAGGCACGTAAAg                                                                                                                                                                                                    <  1:209873‑M2/107‑1 (MQ=255)`  
                                                                                         `ccgcactactacgctgacatctttagcgtaatggcaagtgagggcattgaccttgagttcgaagactctggtctgatgcctgacaccaaggacgtaatccgcatcctgcaagcgcgtatctatgagcaattaacgattgacctctgggaagacgcagaagacttgctcaatgagtacttggaggaagtcgaggaAGTCGCACGACAGAAAGAAATTGACCTCGCTAAGGCCCGTAAAGAACGTCACGAGGGGCGCTTAgaggcacgcagattcaaacgtctgtctcttatacacatctccg                                                                                                                                                                                                    >  1:396058‑M2/195‑280 (MQ=255)`  
                                                                                           `gcactactacgctgacatctttagcgtaatggcaagtgagggcatttaccttgagttcgaagactctggtctgatgcctgacaccaaggacgtaatcagcatccggcaagcgcgtatctatgagcaattaacgattgaccgctggtaagacgcagaagacttgctcaatgaatacttggaggaagtcgaggaAGTCGCACGACAGAAAGAAATTGACCGCGCTAAGGCCCGTAAAGAACGTCACGAGGGGCGCTTAGAGGCACGCAGATTCAAACGTCGCAACCGCAAGGCACGTAAAgcc                                                                                                                                                                                                  <  1:259139‑M2/109‑2 (MQ=255)`  
                                                                                            `cactactacgcttacatctttagcggaatggaaagggagggcattgacattgaattcgaagactctggtctgatgcctgacaccaaggacgtaatccgcatcctgcaagcgcgtatctatgagctattaacgattgacctctgggaagacgcagaagacttgctcaatgaatacttggaggaagtagaggaAGTCGCACGACAGAAAGAAATTGACCGCGCTAAGGCCCGTAAAGAACGTCACGAGGGGCGCTTAGAGGCACGCAGATTCAAACGTCGCAACCGCAAGGCACGTAAAGcac                                                                                                                                                                                                 <  1:312979‑M2/110‑1 (MQ=255)`  
                                                                                            `cactactacgctgacatctttagcgtaatggcaagtgagggcattgaccttgagttcgaagactctggtctgatgcctgacaccaaggacgtaatccgcatcctgcaagcgcgtatctatgagcaattaacgattgacctctgggaagacgcagaagacttgctcaatgaatacttggaggaagtcgaggaAGTCGCACGACAGAAAGAAATTGACCGCGCTAAGGCCCGTAAAGAACGTCACGAGGGGCGCTTAGAGGCACGCAGATTCAAACGTCGCAACCGCAAGGCACGTAAAgcaa                                                                                                                                                                                                 >  1:409414‑M2/192‑300 (MQ=255)`  
                                                                                              `ctactacgctgacatctttagcgtaatggcaagtgagggcattgaccttgagttcgaagactctggtctgatgcctgacaccaaggacgtaatccgcatcctgcaagcgcgtatctatgagcaattaacgattgacctctgggaagacgcagaagacttgctcaatgaatacttggaggaagtcgaggaAGTAGCACGACAGAAAGAAATTGACCGCGCTAAGGCCCGTAAAGAACGTCACGAGGGGCGCTTAGAGGCACGCAGATTCAAACGTCGCAACCGCAAGGCACGTAAAGcacac                                                                                                                                                                                               >  1:301341‑M2/190‑301 (MQ=255)`  
                                                                                                `actccgctgacatctttagcgtaatggcaagtgagggcattgaccttgagttcgaagactctggtctgatgcctgacaccaaggatgtaatccgcatcctgcaagcgcgtatctatgagcaattaacgattgacctctgggaagacgcagaagacttgctcaatgaatacttggaggaagtcgaggaAGTCGCACGACAGAAAGTAATTGACCGCGCTAAGGCCCGTAAAGAACGTCACGAGGGGCGCTAGGAGGCACGAAGAGTCAACCGTGCCACCCCAAAGGCacggaaagaccaaaa                                                                                                                                                                                             >  1:105270‑M2/188‑294 (MQ=255)`  
                                                                                                `actacgctgacatctttagcgtaatggcaagtgatggcattgaccttgagttcgaagactctggtctgatgcctgacaccaaggacgtaatccgcatcctgcaagcgcgtatctatgagcaattaacgattgacctctgggaagacgcataagacttgctcaatgaatacttggcggaattcgaggaAGTCGCCCGACAGAAAGAAATTGACCGCGCTAAGGCCCGTAAAGAACGTCACGAGGGGCGCTTAGAGGCACGCCGATTCAAACGTCGAAACCGCAAGGCACGTAAagcacacca                                                                                                                                                                                             >  1:70871‑M2/188‑299 (MQ=255)`  
                                                                                                `actacgctgacatctttagcgtaatggcaagtgagggcattgaccttgagttcgaagactctggtctgatgcctgacaccaaggacgtaatccgaatcctgcaagcgcgtatctatgagcaattaacgattgacctatgggaagacgcagaagacttgctcaatgaatacttggaggaagtcgaggaAGTCGCACGACAGAAAGAAATTGACCGCGCTAAGGCCCGTAAAGAACGTCACGAGGGGCGCTTAGAGGCACGCAGATTCAAACGTCGCAACCGCAAGGCACGTAAAGCACACaa                                                                                                                                                                                             >  1:281281‑M2/188‑301 (MQ=255)`  
                                                                                                     `gctgacatctttagcgtaatggcaagtgagggcattgaccttgagttcgaagactctggtatgatgcctgacaccaaggacgtaatccgcatcctgcaagcgcgtatctatgagcaattaacgattgacctctgggaagacgcagaagacttgctcaatgaatacttggaggaagtcgaggaAGTCGCACGACAGAAAGAAATTGACCGCGCTAAGGCCCGTAAAGAACGTCACGAGGGGCGCTTAGAGGCACGCAGATTCAAACGTCGCAACCGCAAGGCACGTAAAGCACACAAAGCTa                                                                                                                                                                                        <  1:32109‑M2/119‑1 (MQ=255)`  
                                                                                                            `tctttagcgtaatggcaagtgagggcattgaccttgagttcgaagactctggtctgatgcctgacaccaaggacgtaatccgcatcctgcaagcgcgtatctatgagcaattaacgattgacctctgggaagacgcagaagacttgctcaatgaatacttggaggaagtcgaggaAGTCGCACGACAGAAAGAAATTGACCGAGCTACGGCCCGTAAAGAACGTCACGAGGGGCGCTTAGAGGCACGCAGATTCAAACGTCGCAACCGCAAGGCACGTAAAGCACACAAAGCTaagcgctg                                                                                                                                                                                 >  1:485423‑M2/176‑299 (MQ=255)`  
                                                                                                             `ctttagcgtaatggcaagtgagggcattgaccttgagttcgaagcctctggtctgatgcctgacaccaaggacgtaatccgcatcctgcaagcgcgtatctatgagcaattaacgattgacctctgggaagacgcagaagacttgctcaatgaatacttggaggaagtcgaggaAGTCGCACTACAGAAAGAAATTGACCTCGCTAAGGCCCGTAAAGAACGTCACGATGGGCGCTTAGAGGCACGCAGATTCAAACGTCGCAACCGCAAGGCACGTAAAGCACACAAAGctaagcgacaa                                                                                                                                                                                >  1:40489‑M2/175‑297 (MQ=255)`  
                                                                                                                  `gcgtaatggcaagtgagggcattgaccttgagttcgaagactctggtctgatgcctgacaccaaggacgtaatccgcatcctgcaagcgcgtatctatgagcaattaacgattgacctctgggaagacgcagaagacttgctcaatgaatacttggaggaagtcgaggaAGTCGCACGACAGAAAGAAATTGACCGCGCTAAGGCCCGTAAAGAACGTCACGAGGGGCGCTTAGAGGCACGCAGATTCAAACGTCGAAACCGCAAGGCACGTAAAGCACACAAAGCTAAGCGCGAAAgaaa                                                                                                                                                                           >  1:496385‑M2/170‑300 (MQ=255)`  
                                                                                                                  `gcgtaatggcaagtgagggcattgaccttgagttcgaagactctggtctgatgcctgacaccaaggacgtaatccgcatcctgcaagcgcgtatctatgagaaattaacgattgacctctgggaagacgcagaagacttgctcaatgaatacttggaggaagtcgaggaAGTCGCACGACAGAAAGAACTTGACAGCGCTAAGGCCCGTAAAGAACGTCACGAGGGGCGCTTAGAGGCACGCAGATTCAAACGTCGCAACCGCAAGGCACGTCACGCACACAAAGCTAAGCGCGAACGAAt                                                                                                                                                                           >  1:133591‑M2/170‑301 (MQ=255)`  
                                                                                                                          `gcaagtgaaggaattgaccttgagtgcgaagactctgggctgatgccggacaacaaggacgtaatccgcatcctgaaagcgcgtaaatatgagcaattaaagattgacctatgggaagacgcagaaaacttgaaaaatgaatacttggaggaagtcgaggaAGTCGCACGACAGAAAGAAATTGACCGCGCTAAGGCCCGTAAAGAACGTCACGAGGGGCGCTTAGAGGCACGCAGATTCAAACGTCGCAACCGCAAGGCACGTAAAGCACACAAAGCTAAGCGCGAAAGAATGCTtgctg                                                                                                                                                                   <  1:466624‑M2/140‑1 (MQ=255)`  
                                                                                                                                             `ttgagttcggagactctggtctgatgcctgacaccaaggacgtaatccgcatcctgcaagcgcgtatcgatgagcaattaacgattgacctctgggaagacgcagaagacttgctcaatgaatacttggaggaagtcgaggaAGTCGCACGACAGAAAGAAATTGACCGCGCTAAGGCCCGTAAATAACGTCACGAGGGGCGCTTAGAGGCACGCAGATTCAAACGTCGCAACCGCAAGGCACGTAAAGCACACAAAGCTAAGCGCGAAAGAATGCTTGCTGCGTGTCGATGGGCTGAACg                                                                                                                                                <  1:418626‑M2/159‑1 (MQ=255)`  
                                                                                                                                               `gagttcgaagactctggtctgatgcctgacaccaaggacgtaatccgcatcctgcaagcgcgtatctatgagcaattaacgattgacctctgggaagacgcagaagacttgctcaatgaatacttggaggaagtcgaggaAGTCGCACGACAGAAAGAAATTGACCGCGCTAAGGCCCGTAAAGAACGTCACGAGGGGCGCTTAGAGGCACGCAGATTCAAACGTCGCAACCGCAAGGCACGTAAAGCACACAAAGCTAAGCGCGAAAGAATGCTTGCTGCGTGGCGATGGGCTGAACGTc                                                                                                                                              >  1:447371‑M2/141‑301 (MQ=255)`  
                                                                                                                                                     `gaagactctggtctgatgcgtgacaccaaggaagtaatccgcatcctggaagggcgtatctatgagaaattaaagattgacctatgggaagacgtagaagacttgccaaatgaatacttggaggaagtcaaggaAGTCGCACGACAGAAAGAAATTGACCGCGCTAAGGCCCGTAAAGAACGTCACGAGGGGCGCTTAGAGGCACGCAAATTAAAACGTCGCAACCGCAAGGCACGTAAAGCACACAAAGCTAAGCGCGAAAGAATGCTTGCTGCGTGGCGATGGGCTGAACGTCAAGAAc                                                                                                                                        <  1:153169‑M2/167‑1 (MQ=255)`  
                                                                                                                                                     `gaagacgctggtctgaggcctgacaccaaggacgtaatccgcatcctgcaagcgcgtatctatgagcaattaacgattgacctctgggaagacgcagaagacttgctcaatgaatacttggaggaagtcgaggaAGTCGCACGACAGAAAGAAATTGACCGCGCTAAGGCCCGTAAAGAACGTCACGAGGGGCGCTTAGAGGCACGCAGATTCAAACGTCGCAACCGCAAGGCACGTAAAGCACACAAAGCTAAGCGCGAAAGAATGCTTGCTGCGTGGCGATGGGCTGAACGTCAAGAAc                                                                                                                                        <  1:4433‑M2/167‑1 (MQ=255)`  
                                                                                                                                                             `gggtctgatgcctgacaccaaggacgtaatccgcatcctgaaagcgcgtatctatgagcaattaacgattgacctctgggaagacgcagaagacttgctcaatgaatacttggaggaagtcgaggaAGTCGCACGACAGAAAGAAATTGACCGCGCTAAGGCCCGTAAAGAACGTCACGAGGGGCGCTTAGAGGCACGCAGATTCAAACGTCGCAACCGCAAGGCACGTAAAGCACACAAAGCTAAGCGCGAAAGAATGCTTGCTGCGTGGCGATGGGCTGAACGTCAAGAACGGCGTAAc                                                                                                                                <  1:183384‑M2/175‑1 (MQ=255)`  
                                                                                                                                                              `ggtctgatgcctgacaccaaggacgtaatccgcatcctgcaagcgcgtatctattagcaattaacgattgacctctgggaagacgcagaagacttgctcaatgaatacttggaggaagtcgaggaAGTCGCACGACAGAAAGAAATTGACCGCGCTAAGGCCCGTAAAGAACGTCACGAGGGGCGCTTAGAGGCACGCAGATTCAAACGTCGCAACCGCAAGGCACGTAAAGCACACAAAGCTAAGCGCGAAAGAATGCTTGCTGCGTGGCGATGGGCTGAACGTCAAGAACGGCGTAAcc                                                                                                                               >  1:162349‑M2/126‑301 (MQ=255)`  
                                                                                                                                                                    `atgcctgacaccaaggacgtaagccgaatcctgcaagcgcgtatctatgagcaattaacgattgacctctgggaagacgaagaagacttgctcaatgaatacttggaggaagtcgaggaAGTCGCACGACAGAAAGAAATTGACCGCGCTAAGGCCCGTAAAGAACATCACGAGGGGCGCTTAGAGGCACGCAGATTCAAACGTCGCAACCGCAAGGCACGTAAAGCACACAAAGCTAAGCGCGAAAGAATGCTTGCTGCGTGGCGATGGGCTGAACGTCAAGAACGGCGTAACCATGAgg                                                                                                                         <  1:458438‑M2/182‑1 (MQ=255)`  
                                                                                                                                                                     `agcctgacaccaaggacttaacccgcatcctgcaagcgcgtatctatgagcaattaacgattgaccgctgggaagacgcagaagacttgctcaatgaatacttggaggaagtcgaggaAGTCGCACGACAGAAAGAAATTGACCGCGCTAAGGCCCGTAAAGAACGTCACGAGGGGCGCTTAGAGGCACGCAGATTCAAACGTCGCAACCGCAAGGCACGTAAAGCACACAAAGCTAAGCGCGAAAGAATGCTTGCTGCGTGGCGATGGGCTGAACGTCAAGAACGGCGTAACCATGAGGt                                                                                                                        <  1:317812‑M2/183‑1 (MQ=255)`  
                                                                                                                                                                     `agcctgacaccaaggacgtaatccgcatcctgcaagcgcgtatctatgagcaattaacgattgacctctgggaagacgcagaagacttgctcaatgaatacttggaggaagtcgaggaAGTCGCACGACAGAAAGAAATTGACCGCGCTAAGGCCCGTAAAGAACGTCACGAGGGGCGCTTAGAGGCACGCAGATTCAAACGTCGCAACCGCAAGGCACGTAAAGCACACAAAGCTAAGCGCGAAAGAATGCTTGCTGCGTGGCGATGGGCTGAACGTCAAGAACGGCGTAACCATGAGGt                                                                                                                        <  1:481332‑M2/183‑1 (MQ=255)`  
                                                                                                                                                                            `caccaaggacgtaatccgcatcctgcaagcgcgtatctatgagcaattaacgattgacctctgggaagacgcagaagacttgctcaatgaatacttggaggaagtcgaggaAGTCGCACGACAGAAAGAAATTGACCGCGCTAAGGCCCGTAAAGAACGTCACGAGGGGCGCTTAGAGGCACGCAGATTCAAACGTCGCAACCGCAAGGCACGTAAAGCACACAAAGCTAAGCGCGAAAGAATGCTTGCTGCGTGGCGATGGGCTGAACGTCAAGAACGGCGTAACCATGAGGTAGctggt                                                                                                                 >  1:225108‑M2/112‑299 (MQ=255)`  
                                                                                                                                                                              `ccaaggacgtaatccgcatcctgcaagcgcgtatctatgagcaattaacgattgacctctgggaagacgcagaagacttgctcaatgaatacttggaggaagtcgaggaAGTCGCACGACAGAAAGAAATTGACCGCGCTAAGGCCCGTAAAGAACGTCACGAGGGGCGCTTAGAGGCACGCAGATTCAAACGTAGCAACCGCAAGGCACGTAAAGCACACAAAGCTAAGCGCGAAAGAATGCTTGCTGCGTGGCGATGGGCTGAACGTCAAGAACGGCGTAACCATGAGGTAGCTGTAGa                                                                                                               >  1:75840‑M2/110‑301 (MQ=255)`  
                                                                                                                                                                               `ctatgatgtaatccgcatccgtaaatcgcgtatctatgagcaatgaacgattgaccactgggaagacgcagaagacttgctccatgaatactgggaggaagtcgaggaAGTAGCACGACAGAAAGAAAGTGACCGAGCTAAGGCCCGTAAAGAACGCCACGAGTGGCGCTTAGAGGCACGCAGATTCAAACGTCGCAACCGCAAGGCACGTAAAGCACACAAACCTAAGCGCGAAAGAATGCTTGCGTCGTGGCGATGGGCTGAACGTCAAGAACGGCGTAACCATGAGGTAGCTGTAGAt                                                                                                              <  1:508887‑M2/193‑1 (MQ=255)`  
                                                                                                                                                                               `caaggacgtaatccgcatcctgcaagcgcgtatctatgagcaattaacgattgacctatgggaagacgcagaagacttgctcaatgaatacttggaggaagtcgaggaAGTCGCACGACAGAAAGAAAGTGACCGCGCTAAGGCCCGTAAAGAACGTCACGAGGGGCGCTTAGAGGCACGCAGATTCAAACGTCGCAACCGAAAGGCACGTAAAGCACACAAAGCTAAGCGCGAAAGAATGCTTGCTGCGTGGCGATGGGCTGAACGTCAAGAACGACGTAACCATGAGGTAGCTGTAGAt                                                                                                              <  1:471941‑M2/193‑1 (MQ=255)`  
                                                                                                                                                                               `aaaggacataatccgcatcctgcaagcgcgtatctatgagcaattaacgattgacctctgggaagacgcagaagacttgctcaatgaatacttggaggaagtcgaggaAGTCGCACGACAGAAAGAAATTGACCGCGCTAAGGCCCGTAAAGAACGTCACGAGGGGCGCTTAGAGGCACGCAGATTCAAACGTCGCAACCGCAAGGCACGTAAAGCACACAAAGCTAAGCGCGAAATAATGCTTGCTGCGTGGCGATGGGCTGAACGTCAAGAACGGCGTAACCATGAGGTAGCTGTAGAt                                                                                                              <  1:324841‑M2/193‑1 (MQ=255)`  
                                                                                                                                                                                `aaggacgtaatccccatcctgcaagcgcgtatctatgagaaattaacgattgacctatgggaagacgaagaagacttgctcaatgaatacttggaggaagtcgaggaAGTCGCAGGACAGAAAGAAATTGACCGCGCTAAGGCCCGTAAAGAACGTCACGAGGGGCGCTTAGAGGAACGCAGATTCAAACGTCGCAACCGCAAGGCACGTAAAGCACACAAAGCTAAGCGCGAAAGAATGCTTGCTGCGTGGCGATGGGCTGAACGTCAAGAACGGCGTAACCATGAGGTAGCTGTAGATg                                                                                                             <  1:205093‑M2/194‑1 (MQ=255)`  
                                                                                                                                                                                `aaggacgtaagccgcagccggcaagcactaaactatgagcaattaacgattgacctctgggaagacgcagaagacttgctcaatgaatacttggaggaagtcgaggaAGTCGCACGACAGAAAGAAATTGACCGCGCTAAGGCCCGTAAAGAACGTCACGAGGGGCGCTTAGAGGCACGCAGATTCAAACGTCGCAACCGCAAGGCACGTAAAGCACACAAAGCTAAGCGCGAAAGAATGCTTGCTGCGTGGCGATGGGCTGAACGTCAAGAACGGCGTAACCATGAGGTAGCTGTAGATg                                                                                                             <  1:329759‑M2/194‑1 (MQ=255)`  
                                                                                                                                                                                  `ggacgtaatccgcatcctgcaagcgcgtatctatgagcaattaacgattgacctcttggaagacgcagaagacttgctcaatgaatacttggaggaagtcgaggaAGTCGCACGACAGAAAGAAATTTACCGCGCTAAGGCCCGTAAAGAACGTCACGAGGGGCGCTTAGAGGCACGCAGATTCAAACGTCGCAACCGCAAGGCACGTAAAGCACACAAAGCTAAGCGCTAAAGAATGCTTGCTGCGTGGCGATGGGCTGAACGTCAAGAACGGCGTAACCATGAGGTAGCTGTAGATGTa                                                                                                           >  1:400971‑M2/106‑301 (MQ=255)`  
                                                                                                                                                                                  `ggacgtaatccgcatcctgcaagcgcgtatctatgagcaattaacgattgacctctgggaagacgcagaagacttgctcaatgaatacttggaggaagtcgaggaAGTCGCACTACAGAAAGAAATTGACCGCGCTAGGGCCCGTAAAGAACGTCACGAGGGGCGCTTAGAGGCACGCAGATTCAAACGTCGCAACCGCAAGGCACGTAAAGCACACAAAGCTACGCGCGAAAGAATGCTTGCTGCGTGGCGATGGGCTGAACGTCAAGACCGGCGTAACCATGAGGTAGCTGTAGATGTa                                                                                                           >  1:239334‑M2/106‑301 (MQ=255)`  
                                                                                                                                                                                  `ggacgtaatccacatcctgcaagcgcgtatctatgagcaattaacgattgacctctgggaagacgcaaaagacttgctcaaagaatacttggatgaagtcgaggaAGTCGCACGACAGAAAGAAATTGACCGCGCTAAGGCCCGTAAAGAACGTCACGAGGGGCGCTTAGAGGCACGCAGATTCAAAAGTCCCCACCGCCAGGCACGTAAAGCACACAAACCTAAACCCGAAAAAATGCTTGATGCCGGGCGAATGGCCTAACGTCAAAACCGGCGTAACCATAGGGAAGCTGTCGATGTa                                                                                                           >  1:56417‑M2/106‑301 (MQ=255)`  
                                                                                                                                                                                   `gacgtaatccgcatcctgcaagcgcgtatctatgagcaattaacgattgacctctgggaagacgcagaagacttgctcaatgaatacttggaggaagtcgaggaAGTCGCACGACAGAAAGAAATTGACCGCGCTAAGGCCCGTAAAGAACGTCACGAGGGGCGCTTAGAGGCACGCAGATTCAAACGTCGCAACCGCAAGGCACGTAAAGCACACAAAGCTAAGCGCGAAAGAATGCTTGCTGCGTGGCGATGGGCTGAACGTCAAGAACGGCGTAACCATGAGGTAGCTGTAGATGTAc                                                                                                          >  1:338330‑M2/105‑301 (MQ=255)`  
                                                                                                                                                                                    `acgtaatccgaatactgcaagcgcgtatctatgagcaattaacgattgacctctgggaagacgcagaagacttgctcaatgaatacttggaggaagtcgaggaAGTCGCACGACAGAAAGAAATTGACCGCGCTAAGGCCCGTAAAGAACGTCACGAGGGGCGCTTAGAGGCACGAAGATTCAAACGTCGCAACCGCAAGGCACGTAAAGCACACAAAGCTAAGCGCGAAAGAATGCTTGCTACGTGGCGATGGACTGAACGTCAAGAACGGCGTAACCATGAGGTAGCTGTAGATGTACt                                                                                                         >  1:308194‑M2/104‑301 (MQ=255)`  
                                                                                                                                                                                     `cgtaatccgcatcctgcaagcgcgtatctatgagcaattaacgattgacctctgggaagacgcagaagacttgctcaatgaatacttggaggaagtcgaggaAGTCGAACGACAGAAAGAAATTGACCGCGCTAAGGCCCGTAAAGAACGTCACGAGGGGCGCTTAGAGGCACGCAGATTCAAACGTCGCAACCGCAAGGCACGTAAAGCACACAAAGCTAAGCTCGAAAGAATGCTTGCTGCGTGGCGATGGGCTGAACGTCAAGAACGGCGTAACCATGAGGTAGCTGTAGATGTACTa                                                                                                        <  1:100754‑M2/199‑1 (MQ=255)`  
                                                                                                                                                                                        `aatccgcatcctgcaagcgcgtatctatgagcaattaacgattgacctctgggaagacgcagaagacttgctcaatgaatacttggaggaagtcgaggaAGTCGCACGACAGAAAGAAATTGACCGCGCTAAGGCCCGTAAAGAACGTCACGAGGGGCGCTTAGAGGCACGCAGATTCAAACGTCGCAACCGCAAGGCACGTAAAGCACACAAAGCTAAGCGCGGAAGAATGCTTGCTGCGTGGCGATGGGCTGAACGTCAAGAAAGGCGTAACCATGAGGTAGCTGTAGATGGACTCGga                                                                                                     >  1:414154‑M2/100‑301 (MQ=255)`  
                                                                                                                                                                                          `tccgcatcctgcaagcgcgtatctatgagcaattaacgattgacatctgggaagacgcagaagacttgctcaatgaatacttggaggaagtcgaggaAGTCGCACGACAGAAAGAAATTGACCGCGCTAAGGCCCGTAAAGAACGTCCCGAGGGGCGCTTAGAGGCACGAAGATTCAAACGTCGCAACCGCAAGGCACGTAAAGCACACAAAGCTAAGCGCGAAAGAATGCTTGCTGCGTGGCGATGGGCTGACCgtcaagaacggcgtcaccattaggctttcccttatacacctctccc                                                                                                   >  1:294227‑M2/98‑279 (MQ=255)`  
                                                                                                                                                                                               `atcctgcaagcgcgtatctatgagcaattaactattgacctctgggaagacgcagaagacttgctcaatgaataattggaggaagtcgaggaAGTCGAACGACAGAAAGAAATTGACCGCGCTAAGGCCCGTAAAGAACGTCACGAGGGGCGCTTAGAGGCACGCAGATTCAAACGTCGCAGCCGCAAGGCACGTAAAGCACACAAAGCTAAGCGCGAAAGAATGCTTGCTGCGTGGCGATGGGCTGAACGTCAAGAACGGCGTAACCATGAGGTAGCTGTAGATGTACTAGGAAGAACCa                                                                                              <  1:60527‑M2/209‑1 (MQ=255)`  
                                                                                                                                                                                                `tcctgcaagcgcgtatctatgagcaattaacgattgacctctgggaagacgcagaagacttgctcaatgaatacttggaggaagtcgaggaAGTCGCACGACAGAAAGAAATTGACCGCGCTAAGGCCCGTAAAGAACGTCACGAGGGGCGCTTAGAGGCACGCAGATTCAAACGTCGCAACCGCAAGGCACGTAAAGCACACAAAGCTAAGAGCGAAAGAATGCTTGCTGCGTGGCGATGGGcagaacgtcaagaacggcgtaagcatgcggctgtctcttatacacatcgccgagccca                                                                                             >  1:452634‑M2/92‑273 (MQ=255)`  
                                                                                                                                                                                                  `ctgcaagcgcgtatctatgagcaattaacgattgacctctgggaagacgcagaagacttgctcaatgaatacttggaggaagtcgaggaAGTCGCACGACAGAAAGAAATTGACCGCGCTAAGGCCCGTAAAGAACGTCACGAGGGGCGCTTAGAGGCACGCAGATTCAAACGTCGCAACCGCAAGGCACGTAAAGCACACAAAGCTAAGCGCGAAAGAATGCTTGCTGCGTGGCGATGGGCTGAACGTCAAGAACGGCGTAACCATGAGGTAGCTGTAGATGTACTAGGAAGAACCAATa                                                                                           >  1:390882‑M2/90‑301 (MQ=255)`  
                                                                                                                                                                                                  `ctgaaaacgggtatatatgatcaattaaagaatgacctatgggaagacgaagaagacttgctcaatgaatacttgtatgaagtctaggaAGTCGCACGACAGAAAGAAATTGACCGCGCTAAGGCCCGTAAAGAACGTCACGAGGGGCGCTTAGAGGCACGCAGATTCAAACGTCGCAACCGCAATGCACGTAAAGCACACAAAGCTAAGCGCGAAAGAATGCTTGCTGCGTGGCGATGGGCTGAACGTCAAGAACGGCGTAACCATGAGGTAGCTGTAGATGTACTAGGAAGAACCAATa                                                                                           <  1:251214‑M2/212‑1 (MQ=255)`  
                                                                                                                                                                                                      `aagagctgatctattagcaattaatgattgacctctgggaagacgaagaaaaatgactcaatgaataattggaggaagacgagaaAGGCGCACGACAGAAAGAAATTGACCGCGCTAAGGCGCGAAAAAAACGTCACGAGGGGCGCTTAGAGGCACGCAGATTCAAACGGCGCAACCGCAAGGCACGTAAAGCACACAAAGCTAAGCGCGAAAGAATGCTTGCTTCCTGGCGATGGGCTGAACGTCAAGAACGGCGTAACCATGAGGTAGCTGTAGATGTACTAGGAAGAACCAATAACGc                                                                                       <  1:158359‑M2/216‑1 (MQ=255)`  
                                                                                                                                                                                                       `agcgcgtatctatgagcaattaacgattgacctctgggaagacgcagaagacttgctcaatgaatacttggaggaagtcgaggaAGTCGCACTACAGAAAGAAATTGACCGCGCTAAGGCCCGTAAATAACGTCACGAGGGGCGCTTAGAGGCACGCAGATTCAAACGTCGCAACCGCAAGGCACGTAAAGCACACAAAGCTAAGCGCGAAAGAATGCTTGCTGCGTGGCGATGGGCTGAACGTCAAGAACGGCGTAACCATTAGGTAGCTGTAGATGTACTAGGAAGAACCAATAACGCt                                                                                      >  1:334417‑M2/85‑301 (MQ=255)`  
                                                                                                                                                                                                        `gcgcgtatctatgagcaattaacgattgacctctgggaagacgcagaagacttgctcaatgaatacttggaggaagtcgaggaAGTCGCACGACAGAAAGAAATTGACCGCGCTAAGGCCCGTAAAGAACGTCACGAGGGGCGCTTAGAGGCACGCAGATTCAAACGTCGCAACCGCAAGGCACGTAAAGCACACAAAGCTAAGCGCGAAAGAATGCTTGCTGCGTGGCGATGGGCTGAACGTCAAGAACGGCGTAACCATGAGGTAGCTGTAGATGTACTAGGAAGAACCAATAAcgcct                                                                                     >  1:492436‑M2/84‑299 (MQ=255)`  
                                                                                                                                                                                                           `ggtatctatgagcaattaacgattgacctctgggaagacgcagaagacttgctcaatgaatacttggaggaagtcgaggaAGTCGCACGACAGAAAGAAATTGACCGCGCTAAGGCCCGTAAAGAACGTCACGAGGGGCGCTTAGAGGCACGCAGATTCAAACGTCGCAACCGCAAGGCACGTAAAGCACACAAAGCTAAGCGCGAAAGAATGCTTGCTGCGTGGCGATGGGCTGAACGTCAAGAACGGCGTAACCATGAGGTAGCTGTAGATGTACTAGGAAGAACCAATAACGCTATGc                                                                                  <  1:21965‑M2/221‑1 (MQ=255)`  
                                                                                                                                                                                                           `acaggatatgagcaattaacgattgacctctgggaagacgcagaagacttgctcaatgaatacttggaggaagtcgaggaAGTCGCACGACAGAAAGAAATTGACCGCGCTAAGTCCCGTAAAGAACGTCACGAGGGGCGCTTAGAGGCACGCAGATTCAAACGTCGCAACCGCAAGGCACGTAAAGCACACAAAGCTAAGCGCGAAAGAATGCTTGCTGCGTGGCGATGGGCTGAACGTCAAGAACGGCGTAACCATGAGGTAGCTGTAGATGTACTAGGAAGAACCAATAACGCTATGc                                                                                  <  1:129239‑M2/221‑1 (MQ=255)`  
                                                                                                                                                                                                              `gctcggagatgtgtataagagacattctgggaagacgcagaagacgtgctcaatgaatacttggaggaagtcgaggaAGTCGCACGACAGAAAGAAATTGACCGCGCTAAGGCCCGTAAAGAACGTCACGAGGGGCGCTTAGAGGCACGCAGATTCAAACGTCTCAACCGCAAGGCACGTAAAGCACACAAAGCTAAGCGCGAAAGAATGCTTGCTGCGTGGCGATGGGCTGAACGTCAAGAACGGCGTAACCATGAGGTAGCTGTAGATGTACTAGGAAGAACCAATAACGCTATGctct                                                                               <  1:233294‑M2/224‑1 (MQ=255)`  
                                                                                                                                                                                                               `tctatgagcaattaacgattgacctctgggaagacgcagaagacttgctcaatgaatacttggaggaagtcgaggaAGTCGCACGACAGAAAGAAATTGACCGCGCTAAGGCCCGTAAAGAACGTCACGAGGGGCGCTTAGAGGCACGCAGATTCAAACGTCGCAACCGCAAGGCACGTAAAGCACACAAAGCTAAGCGCGAAAGAATGCTTGCTGCGTGGCGATGGGCTGAACGTCAAGAACGGCGTAACCATGAGGTAGCTGTAGATGTACTAGGAAGAACCAATAACGCTATGCTCTg                                                                              >  1:94224‑M2/77‑301 (MQ=255)`  
                                                                                                                                                                                                               `tctatgagcaattaacgattgacctctggaaagacgcagaagacttgctaaatgaatacttggaggaagtcgaggaAGTCGCACGACAGAAAGAAATTGACCGCGCTAAGGCCCGTAAAGAACGTCACGAGGGGCGCTTAGAGGCACGCAGATTCAAACGTCGCAACCGCAAGGCACGTAAAGCACACAAAGCTAAGCGCGAAAGAATGCTTGCTGCGTGGCGATGGGCTGAACGTCAAGAACGGCGTAACCAtgaggtagctgtagatgtactaggactgtctcttatacacatctccga                                                                              >  1:12449‑M2/77‑278 (MQ=255)`  
                                                                                                                                                                                                                      `gcaattaacgattgacctctgggaagacgcagaagacttgctcaatgaatacttggaggaagtcgaggaAGTCGCACGACAGAAAGAAATTGACCGCGCTAAGGCCCGTAAAGAACGTCACGCGGGGCGCTTAGAGGCACGCAGATTCAAACGTCGCAACCGCAAGGCACGTAAAGCACACAAAGCTAAGCGCGAAAGAATGCTTGCTGCGTGGCGATGGGCTGAACGTCAAGAACGGCGTAACCATGAGGTAGCTGTAGATGTACTAggaagaaccaataacgcctgtctcttatacaca                                                                       >  1:200060‑M2/70‑285 (MQ=255)`  
                                                                                                                                                                                                                       `aaattaacgattgacctctgggaagacgcagaagacttgctcaatgaatacttggaggaaggcgaggaAGTCGCACGACAGAAAGAAATTGACCGCGCTAAGGCCCGTAAAGAACGTCACGAGGGGCGCTTAGAGGCACGCAGATTCAAACGTCGAAACCGCAAGGCACGTAAAGCACACAAAGCTAAGCGCGAAAGAATGCTTGCTGCGTGGCGATGGGCTGAACGTCAAGAACGGCGTAACCATGAGGTAGCTGTAGATGTACTAGGAAGAACCAATAACGCTATGCTCTGGGTCAACa                                                                      <  1:195020‑M2/233‑1 (MQ=255)`  
                                                                                                                                                                                                                        `aattaacgattgaacgctgggaagacgcagaagacttgctcaaggaataattggaggaagtcgaggaAGGCGCACGACAGAAAGAAATTGAACGCGATAAGGCACGTAAAGAACGTCACGAGGGGCGCTTAGAGGCACGCAGATTCAAACGTCGCAACCGCAAGGCACGTAAAGCACACAAAGCTAAGCGCGAAAGAATGCTTGCTGCGTGGCGATGGGCTGAACGTCAAGAACGGCGTAACCATGAGGTAGCTGTAGATGTACTAGGAAGAACCAATAACGCTATGCTCTGGGTCAACAt                                                                     <  1:356327‑M2/234‑1 (MQ=255)`  
                                                                                                                                                                                                                         `attaacgattgacctctgggaagacgcagaagacttgctcaatgaatacttggaggaagtctaggaAGTCGCACGACAGAAAGAAATTGACCGTGCTAAGGCCCGTAAAGAACGTCACGAGGGGCGCTTAGAGGCACGCAGATTCAAACGTCGCAACCGCAAGGCACGTAAAGCACACAAAGCTAATCGCGAAAGAATGCTTGCTGCGTGGCGATGGGCTGAACGTCAAGAACGGCGTAACCATGAGGTAGCTGTAGATGTACTAGGAAGAACCAATAACGCTATGCTCTGGGTCAacact                                                                    >  1:500579‑M2/67‑299 (MQ=255)`  
                                                                                                                                                                                                                         `attaacgagtgacctctgggaagacgcagaagacttgctcaatgaatacgtggaggaagtcgaggaAGTCGCACGACAGAAAGAAATTGTCCGCGCTAAGGCCCGTAAAGAACGTCACGATGGGCGCTTAGAGGCACGCAGATTCAAACGTCGCAACCGCAAGGCACGTAAAGCATACAAAGCTAAGCGCGAAAGAATGCTTGCTGCGTGGCGATGGGCTGAACGTCAAGAACGGCGTAACCATGAGGTAGCTGTAGATGTACTAGGAAGAACCAATAACGCTATGCTCTGGGTCAACATg                                                                    <  1:214950‑M2/235‑1 (MQ=255)`  
                                                                                                                                                                                                                            `aacgattgacctctgggaagacgcagaagacttgctcaatgaatacttggaggaagtcgaggaAGTCGCACGACAGAAAGAAATTGACCGCGCTAAGGCCCGTAAAGAACGTCACGAGGGGCGCTTAGAGGCACGCAGATTCAAACGTCGCAACCGCAAGGCACGTAAAGCACACAAAGCTAAGCGCGAAAGAATGCTTGCTGCGTGGCGATGGGCTGAACGTCAAGAACGGCGTAACCATGAGGTAGCTGTAGATGTACTAGGAAGAACCAATAACGCTATGCTCTGGGTCAACATGTtc                                                                 <  1:489097‑M2/238‑1 (MQ=255)`  
                                                                                                                                                                                                                            `aacgattgacctctgggaaaacgcagaatgctttctcaatgaatacttggaggaagtcgaggaAGTCGCACGACAGAAAGAAATTGACCGCGCTAAGTCCCGTAAAGAACGTCACGAGGGGCGCTTAGAGGCACGCAGATTCAAACGTCGCAACCGCAAGGCACGTAAAGCACACAAAGCTAAGCGCGAAAGAATGCTTGCTGCGTGGCGATGGGCTGAACGTCAAGAACGGCGTAACCATGAGGTAGCTGTAGATGTACTAGGAAGAACCAATAACGCTATGCTCTGGGTCAACATGTtc                                                                 <  1:255131‑M2/238‑1 (MQ=255)`  
                                                                                                                                                                                                                              `cgattgacctctgggaagacgcagaagacttgctcaatgaatacttggaggaagtcgaggaAGTCGCACGACAGAAAGAAATTGACCGCGCTAAGGCCCGTAAAGAACGTCACGAGGGGCGCTTAGAGGCACGCAGATTCAAACGTCGAAACCGCAAGGCACGTAAAGCACACAAAGCTAAGCGCGAAAGAATGCTTGATGCGTGGCGATGGGCTGAACGTCAAGAACGGCGTAACCATGAGGTAGCTGTAGATGTACTAGGAAGAACCAATAACGCTATGCTCTGGGTCAACATGTtatc                                                               >  1:85544‑M2/62‑301 (MQ=255)`  
                                                                                                                                                                                                                              `cgatggacctctggtaagaagaagaaaacttgctcaatgaatactgggaggaagtcgaggaAGTCGAATGACAGAAAGAAATTGGCCGCGCTAAGGCCCGTAAAGAACGTCACGAGGGGCGCTTAGAGGCACGCAGATTCAAACGTCGCAACCGCAAGGCACGTAAAGCACACAAAGCTAAGCGCGAAAGAATGCTTGCTGCGTGGCGATGGGCTGAACGTCAAGAACGGCGTAACCATGAGGTAGCTGTAGATGTACTAGGAAGAACCAATAACGCTATGCTCTGGGTCAACATGTtctc                                                               <  1:350734‑M2/240‑1 (MQ=255)`  
                                                                                                                                                                                                                                 `ttgacctcggggaagacgcagaagacttgctcaatgaagacttggaggaagtcgaggaAGTCGCACGACAGAAAGAAATGGACCGCGCTAAGGCCCGTAAAGAACGTCACGAGGGGCGCTTAGAGGCACGCAGATTCAAACGTCGCAACCGCAAGGCACGTAAAGCACACAAAGCTAAGCGCGAAAGAATGCTTGCTGCGTGGCGATGGGCTGAACGTCAAGAACGGCGTAACCATGAGGTAGCTGTAGATGTACTAGGAAGAACCAATAACGCTATGCTCTGGGTCAACATGTTCTCTgg                                                            <  1:438417‑M2/243‑1 (MQ=255)`  
                                                                                                                                                                                                                                     `cctctgggaagacgcagaagacttgctcaatgaatacttggaggaagtcgaggaAGTCGCACGACAGAAATAAATTGACCGCGCTAAGGCCCGTAAAGAACGTCACGAGGGGCGCTTAGAGGCACGCAGATTCAAACGTCGCAACCGCAAGGCACGTAAAGCACACAAAGCTAAGCGCGAAAGAATGCTTGCTGCGTGGCGATGGGCTGAACGTCAAGAACGGCGTAACCATGAGGTATCTGTAGATGTACTAGGAAGAACCAATAACGCTATGCCCTGGGTCAACATGTTCTCTGGGGAc                                                        >  1:147522‑M2/55‑301 (MQ=255)`  
                                                                                                                                                                                                                                     `cctctgggaagacgcagaagacttgctcaatgaatacttggaggaagtcgaggaAGTCGCACGACAGAAAGAAATTGACCGCGCTAAGGCCCGTAAGGAACGTCACGAGGGGCGCTTAGAGGCACGCAGATTCAAACATCGCAACCGCAAGGCACGTAAAGCACACAAAGCTAAGCGCGAAAGAATGCTTGCTGCGTGGCGATGGGCTGAACGTCAAGAACGGCGTAACCCTGAGGTAGCTGTAGATGTACTAGGACGAACCAATAACGCTATGCCCTGGGTCAACCTGTTCCCTGGGGAc                                                        >  1:229993‑M2/55‑301 (MQ=255)`  
                                                                                                                                                                                                                                     `cctctgggaagacgcagaagacttgctcaatgaatacttggaggaagtcgaggaAGTCGCACGACAGAAAGAAATTGACCGCGCTAAGGCCCGTAAAGAACGTCACGAGGGGCGCTTAGAGGCACGCAGATTCAAACGTCGCAACCGCACGGCACGTAAAGCACACACAGCTAAGCGCGAAAGAATGCTTGCTGCGTGGCGATGGGCTGAACGTCAAGAACGGCGTAACCATGAGGTAGCTGACGATGTACTAGTAAAAACCAATAACGCTAAGccctgggtcaacatccgtctcttatac                                                        >  1:433636‑M2/55‑288 (MQ=255)`  
                                                                                                                                                                                                                                            `gaagacgcagaagacttgctcaatgaatacttggaggaaggcgaggaAGTCGCAAGACAGAAAGAAAATGACCGCGATAAGGCCCGTAAAGAACGTCACGAGGGGCGCTTAGAGGCACGCAGATTCAAACGTCGCAACCGCAAGGCACGTAAATCACACAAAGCTAAGCGCGAAAGAATGCTTGCTGCGTGGCGATGGGCTGAACGTCAAGAACGGCGTAACCATGAGGTAGCTGTAGATGTACTAGGAAGAACCAATAACGCTATGCTCTGGGTCAACATGTTCTCTGGGGACTTTAAgg                                                 <  1:287218‑M2/254‑1 (MQ=255)`  
                                                                                                                                                                                                                                            `caggacgcagaaaaaatgctcactgaatacttggaggaagtctaggaAGTCGCACGACAGAAAGAAATGGACGGCGTGTAGGCGCGTGAATAACGTCACGAATGGAAGTTAGAGGCACGCAGATTCAAACGTCGCAACCGCAAGGCACGTAAAGCACACACAGCTAAGCGCGAAAGAATGCTTTCTTCGTGGGGATGTGCTGAACGTCAAGAACGGCGTAACCATGAGGTAGCTGTAGATGTACTAGGAAGAACCAATAACGCTATGCTCTGGGTCAACATGTTCTCTGGGGACTTTAAgg                                                 <  1:110422‑M2/254‑1 (MQ=255)`  
                                                                                                                                                                                                                                              `cagcgcagaagacttgctcaatgaatacttggaggaagtcgaggaAGTCGCACGACAGAAAGAAATTGACCGCGCTAAGGCCCGTAAAGAACGTCACGAGGGGCGCTTAGAGGCACGCAGATTCAAACGTCGCAACCGCAAGGCACGTAAAGCACACAAAGCTAAGCGAGAAAGAATGCTTGCTGCGTGGCGATGGGCTGAACGTCAAGAACGGCGTAACCATGAGGGAGCTGTAGATGTACTAGGAAGAACCAATAAAGCTATGCTCTGGGTCAACATGTTCTCTGGGGACTTTAAGgcg                                               >  1:38137‑M2/46‑301 (MQ=255)`  
                                                                                                                                                                                                                                                        `gacttgctcaatgaatacttggaggaagtcgagtaAGTCGCACGCCAGAAAGAAATTTACCGCGCTAATTCCCGTAAAGAACGTCACGCGGGGCGCTTAGAGGCACGCAGATTCAAACGTCGCAACCGCAAGGCACGTAAAGCACACAAATCTAAGCGCGAAAGAATGCTTGCTGCGTGTAGATTGGCTGAACGTCAAGAACGGCGTAACCATGCGGTCGCTGTAGATGTACTAGGAAGAACCAATAACGCTATGCTCTGGGTCCACATGTTCTCTGTGGACTTTAAGGCTCTTGAGGAAc                                     >  1:178404‑M2/36‑301 (MQ=255)`  
                                                                                                                                                                                                                                                            `tgctaaatgaatacttggaggaagtcgaggaAGTCGCACGACAGAAAGAAATTGACCGCGCTAAGGCCCGTAAAGAACGTCACGAGGGGCGCTTAGAGGCACGCAGATTCAAACGTCGCAACCGCAAGGCACGTAAAGCACACAAAGCTAAGCGCGAAAGAATGCTTGCTGCGTGGCGATGGGCTGAACGTCAAGAACGGCGTAACCATGAGGTAGCTGTAGATGTACTAGGAAGAACCAATAACGCTATGCTCTGGGTCAACATGTTCTCTGGGGACTTTAAGGCGCTTGAGGAACGAAt                                 <  1:272955‑M2/270‑1 (MQ=255)`  
                                                                                                                                                                                                                                                               `tcaatgaatacttggaggaagtcgaggaAGTCGCACGACAGAAAGAAATTGACCGCGCTAAGGCCCGTAAAGAACGTCACGAGGGGCGCTTAGAGGCACGCAGATTCAAACGTCGCAACCGCAAGGCACGTAAAGCACACAAAGCTAAGCGCGAAAGAATGCTTGCTGCGTGGCGATGGGCTGAACGTCAAGAACGGCGTAACCATGAGGTAGCTGTAGATGTACTAGGAAGAACCAATAACGCTATGCTCTGGGTCAACATGTTCTCTGGGGACTTTAAGGCGCTTGAGGAACGAATcgc                              >  1:29784‑M2/29‑301 (MQ=255)`  
                                                                                                                                                                                                                                                                    `gaatacttggaggaagtcgaggaAGTCGCACGACAGAAAGAAATTGACCGCGCTAAGGCCCGTAAAGAACGTCACGAGGGGCGCTTAGAGGCACGCAGATTCAAACGTCGCAACCGCAAGGCACGTAAAGCACACAAAGCTAAGCGCGAAAGAATGCTTGCTGCGTGGCGATGGGCTGAACGTCAAGAACGGCGTAACCATGAGGTAGCTGTAGATGTACTAGGAAGAACCAATAACGCTATGCTCTGGGTTAACATGTTCTCTGGGGACTTTAAGgcgcttgaggaacctgtctcttata                         >  1:8935‑M2/24‑289 (MQ=255)`  
                                                                                                                                                                                                                                                                         `cttggaggaagtcgaggaAGTCGCACGACAGAAAGAAATTGACCGCGCTAAGGCCCGTAAAGAACGTCACGAGGGGCGCTTAGAGGCACGCAGATTCAAACGTCGCAACCGCAAGGCACGTAAAGCACACAAAGCTAAGCGCGAAAGAATGCTTGCTGCGTGGCGATGGGCTGAACGTCAAGAACGGCGTAACCATGAGGTAGCTGTAGATGTACTAGGAAGAACCAATAACGCTATGCTCTGGGTAAACATGTTCTCTGGGGACTTTAAGGCGCTtgaggaacgaatctgtctcttataa                    >  1:267136‑M2/19‑289 (MQ=255)`  
                                                                                                                                                                                                                                                                           `tggaggaagtcgaggaAGTCGCAAGACAGAAAGAAATTGACCGCGCTAAGGCCCGGAAAGAACGTCACGAGGGGCGCTTAGAGGAACGCAGATTCAAACGTCGCAACCGCAAGGCACGTAAAGCACACAAAGCTAAGCGCGAAAGAATGCTTGCTGCGTGGCGATGGGCTGAACGTCAAGAACGGCGTAACCATGAGGTAGCTGTAGATGTACTAGGAAGAACCAATAACGCTATGCTCTGGGTCAACATGTTCTCTGGGGACTTTAAGGCGCTTGAGGAACGAATCGCGCTGCACTGGCg                  <  1:262765‑M2/285‑1 (MQ=255)`  
                                                                                                                                                                                                                                                                                 `aagtcgaggaAGTCGCACGACAGAAAGAAATTGACCGCGCTAAGGCCCGTAAAGAACGTCACGAGGGGCGCTTAGAGGCACGCAGATTCAAACGTCGCAACCGCAAGGCACGTAAAGCACACAAAGCTAAGCGCGAAAGAATGCTTGCTGCGTGGCGATGGGCTGAACGTCAAGAACGGCGTAACCATGAGGTAGCTGTAGATGTACTAGGCAGAACCAATAACGCTATGATCTGGGTCAATATGTTATCTGGGGACTTTAAGGCGCTTGAGGTACGAATCGTGCTGCACTGGCGTAAtgt            >  1:423205‑M2/11‑300 (MQ=255)`  
                                                                                                                                                                                                                                                                                   `gtcgaggaAGTCGCACGACAGAAAGAAATTGACCGCGCTAAGGCCCGTAAAGAACGTCACGAGGGGCGCTTAGAGGCACGCAGATTCAAACGTCGCAACCGCAAGGCACGTAAAGCACACAAAGCTAAGCGCGAAAGAATGCTTGCTGCGTGGCGATGGGCTGAACGTCAAGAACGGCGTAACCATGAGGTAGCTGTAGATGTACTAGGAAGAACCAATAACGCTATGCTCTGGGTCAACATGTTCTCTGGGGACTTTAAGGCGCTTGAGGAACGAATCGCGCTGCACTGGCGTAATGCTg          >  1:284293‑M2/9‑301 (MQ=255)`  
                                                                                                                                                                                                                                                                                   `gtcgaggaAGTCGCACGACAGAAAGAAATTGACCGCGCTAAGGACCGTAAAGAACGTCACGAGGGGCGCTTAGAGGCACGAAGATTCAAACGTCGCAACCGCAAGGCACGTAAAGCACACAAAGCTAAGCGCGAAAGAATGCTTGCTGCGTGGCGATGGGCTGAACGTCAAGAACGGCGTAACCATGAGGTAGCTGTAGATGTACTAGGAAGAACCAATAACGCTATGCTCTGGGTCAACATGTTCTCTGGGGACTTTAAGGCGCTTGAGGAACGAATCGCGCTGCACCGGCGTCAtgcct          >  1:373220‑M2/9‑299 (MQ=255)`  

                                                                                                                                                                                                                                                                                           `|`                                                                                                                                                                                                                                                                                                              
`ACAATGATGCCCATTATAAATACGCTGAGATTGCAAGCGACATCATTGATTGCAATTCGGATGAGCATGATGAGTTAACTGGATGGGATGGTGAGTTTGTTGAAACTTGTAAACTAATCCGCAAGTTCTTTGAGGGCATCGCCTCATTCGACATGCATAGCGGGAACATCATGTTCTCAAATGGAGACGTACCATACATCACCGACCCGGTATCATTCTCGCAGAAGAAAGACGGTGGCGCATTCAGCATCGACCCTGAGGAACTCATCAAGGAAGTCGAGGAAGTCGCACGACAGAAAGAAATTGACCGCGCTAAGGCCCGTAAAGAACGTCACGAGGGGCGCTTAGAGGCACGCAGATTCAAACGTCGCAACCGCAAGGCACGTAAAGCACACAAAGCTAAGCGCGAAAGAATGCTTGCTGCGTGGCGATGGGCTGAACGTCAAGAACGGCGTAACCATGAGGTAGCTGTAGATGTACTAGGAAGAACCAATAACGCTATGCTCTGGGTCAACATGTTCTCTGGGGACTTTAAGGCGCTTGAGGAACGAATCGCGCTGCACTGGCGTAATGCTGACCGGATG  >  NC_001604.1/2454‑3037`

  

Alignment Legend

Aligned base mismatch/match (shaded by quality score): `ATCG/ATCG` `< 3 ≤` `ATCG/ATCG` `< 9 ≤` `ATCG/ATCG` `< 19 ≤` `ATCG/ATCG` `< 34 ≤` `ATCG/ATCG` `< 38 ≤` `ATCG/ATCG`

Unaligned base: `atcg`    Masked matching base: `atcg`    Alignment gap: `‑`    Deleted base: `‑`

Reads not counted as support for junction

read\_name Not counted due to insufficient overlap past the breakpoint.

read\_name Not counted due to not crossing MOB target site duplication.
